# Characteristics of Fertility Transition Response to the Cumulative Effective Low Temperature in a Two-Line Male Sterile Rice Cultivar

**DOI:** 10.1186/s12284-021-00514-8

**Published:** 2021-08-03

**Authors:** Linxuan Wu, Huazhen Rong, Chun Chen, Xin Lin, Jiafeng Wang, Wuming Xiao, Cuihong Huang, Tao Guo, Hui Wang, Ming Huang

**Affiliations:** National Engineering Research Center of Plant Space Breeding, South China Agricultural University, Guangzhou, 510642 People’s Republic of China

**Keywords:** Two-line male sterile rice, Fertility transition, Critical sterile inducing temperature, Effective low temperature

## Abstract

**Background:**

Photo-thermo-sensitive genic male sterile (PTGMS) rice (*Oryza sativa* L.) is usually considered two-line male sterile rice because of its dual-purpose in two-line hybrid rice system: under short days and low temperatures, it is fertile and used for self-propagation, but under long days and high temperatures, it is sterile and used for hybrid seed production. Therefore, photoperiod and temperature conditions are extremely important for the fertility transition of two-line male sterile rice. In recent years, there have been frequent occurrences of abnormally low-temperature (ALT) resulting in failure of two-line hybrid rice seed production. The daily average temperature (DAT) during ALT events is sometimes higher than the critical sterility-inducing temperature (CSIT) of two-line male sterile rice, of which the night temperature is lower than the CSIT. DAT has been traditionally used as the single indicator of pollen fertility transition, but it is unknown why the fertility of two-line male sterile rice in seed production restored fertility under ALT conditions.

**Results:**

For Hang93S (H93S), a newly released PTGMS line, we hypothesized fertility transition is determined mainly by the cumulative effective low temperature (ELT) and only a certain duration of low temperature is required every day during the fertility-sensitive period. This study simulated ALTs where the DAT was higher than the CSIT while some segments of night temperature were lower than the CSIT. The results showed H93S exhibited a fertility transition to varying degrees. Moreover, fertility was restored under simulated ALT conditions and pollen fertility increased with increasing cumulative ELT, indicating that the fertility transition was affected primarily by the cumulative ELT. Results also indicated that pollen fertility increased as the number of treatment days increased.

**Conclusions:**

The fertility transition is caused mainly by the cumulative ELT. In two-line male sterile rice breeding, the effects of day length, ALT at night, and continuous response days should be considered together. The present study provides new insight into fertility transition so breeders can more effectively utilize the two-line male sterile rice, H93S, in breeding programs.

**Supplementary Information:**

The online version contains supplementary material available at 10.1186/s12284-021-00514-8.

## Background

Early investigations of hybrid rice (*Oryza sativa* L.) cultivars developed via the three line system involving a cytoplasmic male sterile A-line, maintainer B-line and restorer R-line (Yuan and Tang 1999), demonstrated a 10–30% yield advantage of the hybrid cultivars over conventional rice cultivars which was attributed to heterosis (Virmani et al. 1982). Additional success was achieved by introducing a two-line hybrid rice system (Chen et al. 2011) in which the male parent is fertile under low temperatures and short days but sterile under high temperatures and long days. The discovery of the photoperiod-sensitive genic male sterility (PGMS) rice line, NongKen58S, in Hubei, China, revolutionized the use of rice heterosis (Shi 1985; He et al. 2007). PGMS and the more recently developed PTGMS lines are the two major types of male sterile rice lines used in two-line hybrid system (Chen and Xiao 2010; Zhou et al. 2012; Chang et al. 2016). With a better understanding of the photoperiod- and temperature-induced fertility transitions associated this system, various genetic models or hypotheses have been proposed for producing two-line male sterile rice, substantially expediting the pace of the breeding process and the development of hybrid combinations (Zhou et al. 2014; Arasakesary et al. 2015; Wu et al. 2016; Barman et al. 2019; Chen et al. 2020). The planting area of two-line hybrid rice is continually increasing and has gradually approached that of three-line hybrid rice, thus playing an increasingly important role in ensuring food security in China (Yuan 2014). Nonetheless, the two-line system has inherent problems associated with the hybrid seed production process, with fertility being affected by the photoperiod/thermo- conditions (Chen et al. 2011). The fertility of two-line male sterile rice can be restored under low temperatures and short day length, resulting in self-propagation, while high temperatures with long days maintain male sterility and can be used for hybrid seed production (Ding et al. 2012; Zhou et al. 2012; Chang et al. 2016; Wang and Deng 2018). However, owing to this unique fertility transition relationship, specialized breeding procedures are required, including the use of artificial climate chambers, the proper timing of planting, and the selection of suitable locations (DeGuzman and Oard 2019). As such breeding and seed production have been limited, thus hindering further development of two-line hybrid rice. During the breeding process of PTGMS rice lines, the CSIT drifts upward (e.g. the CSIT increased 0.9 °C for PeiAi64S, and 2.0 °C for Hengnong S-1) after 3 to 4 generations of propagation (Lei et al. 2014). It was proposed that heterozygosity for the minor male sterility-related gene(s) may cause CSIT drift in two-line male sterile rice. Thus, for two-line hybrid seed production, it is necessary to fully consider the photoperiod and temperature to evaluate the fertility of female parents when determining the location and period for hybrid seed production (Chen et al. 2011; Lei et al. 2014).

In recent years, abnormal-low temperature weather events have been frequent, causing the failure of two-line hybrid rice seed production (Lei and Chen 2015). ALT refers to the DAT being higher than the CSIT, and some segments of the nighttime temperature being lower than the CSIT. For instance, Wan’ning (110.29°N, 18.84°E), which located north of Lingshui (109.95°N, 18.57°E), is generally considered an area that is not suitable for two-line sterile line propagation. However, we observed successive propagation of two-line male sterile rice in the Dongxing Farm Station of Wan’ning for seven consecutive years primarily due to the shift of the sensitive period to occur around mid-February, during which the nighttime low temperatures range from 20.0 °C to 23.0 °C and the daytime temperatures range from 25.0 °C to 28.0 °C, according to historical weather data from 2012 to 2018. According to our field measurements, this region occasionally experienced daytime temperatures ranging from 29.0 °C to 30.0 °C, and the DAT ranged from 21.0 °C to 24.0 °C.

These variations in temperature represent the primary bottleneck limiting reliable hybrid seed production via a two-line male sterile rice system (Chen and Xiao 2010). Chen et al. (2011) proposed that the thermo-sensitive dual-purpose genic male sterile rice must be exposed to a certain amount of cumulative low temperature to change the fertility. However, the mechanism through which low temperature exposure affecting the fertility transition of sterile lines has not been elucidated. Further, according to our observations for the past several years, the DAT of ALT events was sometimes higher than the CSIT of two-line male sterile rice, which fails to explain why the fertility was restored for the sterile line during the hybrid seed production. Thus, we hypothesized that the fertility transition of the two-line male sterile rice during the fertility-sensitive period is determined mainly by the cumulative effective low temperature (ELT) and that only a certain cumulative of low temperature exposure is needed every day during the fertility-sensitive period during the stage IV to VI of the young panicle differentiation (Chen et al. 2011). When a certain cumulative ELT is reached, the sterile line transitions to a fertility line. In the present study, we used the PTGMS lines H93S and PeiAi64S (PA64S) to investigate the photo-thermo-response characteristics and the correlation between the number of effective treatment days for fertility transition and the cumulative ELT (ΣT_Δ_). The objectives of this study were to: 1) validate our hypothesis that the fertility transition is determined mainly by the cumulative ELT and a certain duration of low temperature exposure is needed every day during the fertility-sensitive period, and 2) characterize and explore the regulation of ELT during the fertility transition in two-line male sterile rice.

## Materials and Methods

### Plant Materials

H93S, a newly released PTGMS line developed at South China Agricultural University, was derived from the crossing S-25 (a male sterile breeding line derived from C815S/Hefengzhan as the recurrent parent) and M-93 (an inbred line with large panicles and good grain quality selected from the space-radiation-induced mutagenesis of Texianzhan13), followed by backcrossing and selfing (Huang et al. 2018). Further, PA64S, which was used as a control, is also a PTGMS line and has been the most successful female line with the greatest planting area among early two-line hybrid rice in China (Hu et al. 2015).

### Planting, Transplanting and Sampling

H93S and PA64S were sown every 10 d from March 13 through June 11, 2019, for a total of 10 sowing cycles. For each sowing, at least 100 seedlings (30-d old) spaced 12.5 cm × 25 cm were transplanted into fields at the South China Agricultural University Experimental Station, Guangzhou, China (23.13°N, 113.36°E). Plants whose distance between the ligule of the flag leaf and that of the next leaf on the main-culm was about 2.0 cm, were transplanted into the plastic buckets (each bucket carrying three plants was used per treatment, and each treatment was replicated three times) for use in the treatments. At the end of the treatment, the tillers whose distance between the ligule of flag leaf and that of the next leaf on the culm was about 1.0 cm (Chen et al. 2011) were marked, after which the plants were moved back to the field for normal growth under natural conditions (Zhang et al. 2011; Bai et al. 2015).

### Determination of the CSIT

The temperature and daily photoperiod were selected according to the Ministry of Agriculture Standard of China (NY/T1215–2006) (Sun et al. 2006) with slight modifications (for photoperiods of 11.5 h and 12.5 h, the treatment 23.0 °C was applied via an artificial climate chamber; for photoperiods of 13.5 h and 14.5 h, treatments involved 22.5 °C); additional details are provided in Table 1. The temperature was constant, and the light intensity was 30,000 Lx during the day and 0 Lx at night.

**Table 1:** Fertility results in response to light-temperature combinations for the CSITs of H93S and PA64S

Daily photoperiod time (h)	Temperature (°C)	H93S		PA64S	
		PFP (%)	SSP (%)	PFP (%)	SSP (%)
11.5	28.0	0	0	0	0
	24.0	0.01 ± 0	0	78.31 ± 5.27	39.54 ± 7.32
	23.0	50.51 ± 5.53	17.84 ± 4.08	60.37 ± 4.86	22.42 ± 2.94
12.5	28.0	0	0	0	0
	24.0	0	0	0.34 ± 0.29	0
	23.0	8.21 ± 1.81	2.54 ± 0.92	25.25 ± 4.61	15.89 ± 0.22
13.5	28.0	0	0	0	0
	24.0	0	0	0	0
	23.0	0	0	1.19 ± 0.49	0.24 ± 0.22
	22.5	27.27 ± 2.47	12.22 ± 2.84	31.89 ± 10.46	16.93 ± 3.89
14.5	28.0	0	0	0	0
	24.0	0	0	0	0
	23.0	0	0	0	0
	22.5	8.91 ± 3.55	3.72 ± 1.40	16.94 ± 4.11	9.85 ± 1.39

The temperature and daily photoperiod were selected according to the Ministry of Agriculture Standard of China (NY/T1215–2006), with modifications. (The modifications included the following additional treatments: an 11.5 h photoperiod at 23.0 °C, a 12.5 h photoperiod at 22.5 °C, a 13.5 h photoperiod at 22.5 °C, and a 14.5 h photoperiod at 22.5 °C.) Abbreviations are: PFP: pollen fertility percentage; SSP: self-seed setting percentage. All data are expressed as the means ±the standard errors

### Effects of the ELT

Xu et al. (2002) released a protocol of photo-treatment and thermo-treatment settings to simulate low temperature conditions. The temperature settings were as follows: 05:00–11:00 at 25.0 °C, 11:00–17:00 at 26.0 °C, 17:00–23:00 at 23.5 °C, and 23:00–05:00 (the next day) at 19.5 °C, with an average of 23.5 °C during the day. The light intensity settings were as follows: 06:30–08:00 at 5000 Lx, 08:00–18:00 at 20,000 Lx, 18:00–20:00 at 5000 Lx, and 20:00–06:00 at 0 Lx. On the basis of the above settings, the low temperature varied for different durations as outlined in Table 2. The light intensity settings (simulating sunlight) and temperature settings were not synchronized and were mutually exclusive. As shown in Table 2, the temperature was set above the CSIT (T_c_) for t_1_ and below T_c_ for t_2_ to simulate ALT (especially low temperatures at night) during the sensitive period, and T_Δ_ is the value of the ELT, i.e., the difference between actual treatment temperature (T) and the T_c_. T_Δ_ was set to two different values: T_Δ1_ = 2.5 °C (Supplementary Table S1) and T_Δ2_ = 2.0 °C (Supplementary Table S2). T_2_ varied from 7 h to 17 h (t_2_ = 7 h, 9 h, 11 h, 13 h, 15 h and 17 h), and together with the different selected T_Δ_, t_1_, and t_2_ values, 12 treatments were established in which t_1_ = 24 h-t_2_–6 h. For example, assuming that the CSIT of H93S is 23.0 °C (i.e., T_c_ = 23.0 °C) and that the T_Δ1_ = 2.5 °C, t_1_ = 11 h, and t_2_ = 7 h, the DAT (24.15 °C, Supplementary Table S1) can be calculated. The two-line male sterile rice during the fertility-sensitive period showed different degrees of sensitivity to the ΣT_Δ_, which relates to our hypothesis concerning the effect of cumulative ELT. The ΣT_Δ_ can be calculated via ${\sum}_1^d{T}_{\Delta  }$, where *d* is the number of treatment days. The images in Supplementary Fig. S1 show the treatments and facilities used for these studies.

**Table 2:** The temperature and light conditions for the ΣT_∆_ for each day

Temperature conditions		Light conditions	
Treatment time (h)	Temperature (°C)	Time range	Light intensity (Lx)
6: (05:00–11:00)	25.0 °C	06:30–08:00	5000
t_1_: 11:00-(11:00 + t_1_)	26.0 °C	08:00–18:00	20,000
t_2_: (11:00+ t_1_)-05:00	T_c_-T_Δ_	18:00–20:00	5000
		20:00–06:00	0

T_c_ is the value of the CSIT, the T_c_ for H93S is 23.0 °C and for PA64S is 24.0 °C; T_Δ_ is the value of the ELT (i.e., the difference between actual treatment temperature T and T_c_), the T_Δ_ is set at two levels: T_Δ1_ = 2.5 °C and T_Δ2_ = 2.0 °C; t_1_ and t_2_ are the times at which temperatures above and below the CSIT, respectively, and t_2_ values of 7 h, 9 h, 11 h, 13 h, 15 h and 17 h correspond to t_1_ values 11 h, 9 h, 7 h, 5 h, 3 h and 1 h, respectively, for a total of 18 h (t_1_ + t_2_ + 6 = 24 h); The photoperiod settings (simulating sunlight) and temperature settings were not synchronized and were mutually exclusive. Supplementary Table S1 and S2 show the detailed settings

### Study of the Response to ELT Treatment Days

The T_Δ_ included six different levels (0 °C, 1.0 °C, 1.5 °C, 2.0 °C, 2.5 °C, and 3.0 °C), and at each temperature level, the plants were treated for 4 d, 5 d, 6 d, and 7 d, resulting in 24 treatments (Supplementary Table S3 and Fig. S1). The treatments included natural light with an actual photoperiod of approximately 13.5 h. At 4 d, 5 d, 6 d, and 7 d after treatment, the treated plants in each treatment group were transplanted back to the field.

### Detection of Pollen Fertility and Self-Seed Setting Percentage (SSP)

Anthers of five mature florets from a panicle that had initiated a heading were collected for three consecutive days, and they were subjected to microscopic examination. Pollen grains were stained with 1% potassium iodide solution (I_2_-KI), observed under a microscope, and the numbers of fertile and sterile pollen grains were counted in three fields of view for the same slide. Round and deeply stained pollens were considered fertile and pollen fertility percentage (PFP) was calculated from the number of fertile grains divided by the total number of grains. After sampling was performed, the flowered spikelets from the panicle were removed, after which the panicle was bagged. Two weeks later, the SSP was determined.

## Results

### Photo-Thermo-Response Characteristics of H93S during the Fertility-Sensitive Period and its CSIT

During the fertility-sensitive period, H93S and the control (PA64S) were subjected to various combinations of light and temperature. Afterward, the fertility rates after the treatment were calculated, the results of which are shown in Table 1 and Supplementary Fig. S2. At 23.0 °C, the PFPs of H93S were 0% under a 14.5 h or 13.5 h photoperiod and 8.21% under a 12.5 h photoperiod with a SSP of 2.54%, which increased to 50.51% with an 11.5 h photoperiod with a corresponding SSP of 17.84%. At 22.5 °C, the PFP and SSP of H93S under a 13.5 h photoperiod were significantly higher than those under a 14.5 h photoperiod. The 11.5 h and 12.5 h photoperiod treatments were not tested because under these conditions, two-line male sterile rice generally shows normal fertility, rendering the treatment unnecessary (Chen et al. 2011). At 24 °C, the fertility of H93S slightly fluctuated only under short-photoperiod conditions (11.5 h), with an average PFP of less than 0.01%. At 28 °C, H93S and the control were 100% sterile regardless of the photoperiod. Thus, the CSIT of H93S is 23.0 °C under 13.5 h to 14.5 h photoperiods, which is in agreement with the results of the official report when H93S was released (Huang et al. 2018).

Clearly, at the same temperature in the fertility-restoration temperature range, the fertility of H93S increased as the photoperiod decreased, suggesting that H93S is a photo-thermo-sensitive interaction type. The control, PA64S, also exhibited similar photo-thermo-response characteristics associated with fertility transition, and the CSIT was 24.0 °C under a 13.5 h photoperiod.

### Effects of Cumulative ELT

Both H93S and PA64S were subjected to various ELT treatments (for 7 d under a 13.5 h photoperiod; Table 2, Supplementary Tables S1 and S2) during the fertility-sensitive period to simulate ALT for which the DAT was higher than the CSIT, but the temperature during the night was lower than the CSIT. The results of the fertility assessment are shown in Table 3. Among the 12 temperature treatment combinations, seven presented DATs higher than the CSIT for H93S (T_c_ = 23.0 °C under a 13.5 h photoperiod), and five treatments presented DATs lower than the CSIT. For example, when t_1_ = 11 h and t_2_ = 7 h, two values of ELT (T_Δ_) were used during the night (T_Δ1_ = 2.5 °C, T_Δ2_ = 2.0 °C), and at T_Δ1_, the plants were treated with 20.5 °C (T_c_-T_Δ1_), which is 2.5 °C lower than the CSIT during the nighttime, corresponding to an ΣT_Δ1_ = (T_Δ1_ (2.5 °C)*t_2_ (7 h)*7 d)/24 h = 5.10 °C·d and a DAT of {(6 h*25 °C) + (t_1_ (11 h)*26 °C) + (t_2_ (7 h)*(T_c_ (23 °C)-T_Δ1_ (2.5 °C))}/24 h = 24.15 °C. Similarly, at T_Δ2_, the treatment corresponded to ΣT_Δ2_ = 4.08 °C·d and a DAT of 24.29 °C. These results indicated that the DATs of the two temperature treatments were higher than the CSIT of H93S (23.0 °C), however, owing to the ELT treatment at night, the fertility of H93S restored, as reflected by PFPs of 3.10% and 1.53% and corresponding SSPs of 1.31% and 1.01%, respectively. In the T_Δ1_ and T_Δ2_ treatments under t_1_ = 9 h and t_2_ = 9 h, at t_1_ = 7 h and t_2_ = 11 h, and with respect to T_Δ2_ treatment at t_1_ = 5 h and t_2_ = 13 h, all the DATs were all higher than the CSIT, and H93S showed varying degrees of PFP and SSP. In the remaining five treatments in which the DAT was lower than the CSIT, the PFP and SSP of H93S were higher than those in the aforementioned seven treatments, with the greatest PFP of 42.36% and the greatest SSP of 10.06% in the T_Δ1_ treatment at t_1_ = 1 h and t_2_ = 17 h, respectively.

**Table 3:** The PFPs and SSPs of H93S and PA64S after 7 d of treatment under different ΣT_∆_ conditions and a 13.5 h photoperiod

PTGMS lines	T_Δ_ (°C)	PFP (%)	SSP (%)	PFP (%)	SSP (%)	PFP (%)	SSP (%)	PFP (%)	SSP (%)	PFP (%)	SSP (%)	PFP (%)	SSP (%)
		t_1_ = 11 h, t_2_ = 7 h; T_d_ = 24.15 °C		t_1_ = 9 h, t_2_ = 9 h; T_d_ = 23.69 °C		t_1_ = 7 h, t_2_ = 11 h; T_d_ = 23.23 °C		t_1_ = 5 h, t_2_ = 13 h; T_d_ = 22.77 °C		t_1_ = 3 h, t_2_ = 15 h; T_d_ = 22.31 °C		t_1_ = 1 h, t_2_ = 17 h; T_d_ = 21.85 °C	
**H93S**	T_c_ = 23.0 °C T_Δ1_ = 2.5 °C	3.10 ± 0.21	1.31 ± 0.11	5.41 ± 0.60	2.05 ± 0.37	12.50 ± 2.07	3.01 ± 1.07	15.96 ± 0.47	3.44 ± 0.37	36.12 ± 0.28	7.84 ± 0.36	42.36 ± 0.35	10.06 ± 0.42
∑T_∆_/°C·d		5.10		6.56		8.02		9.48		10.94		12.40	
**PA64S**	T_c_ = 24.0 °C T_Δ1_ = 3.5 °C	5.27 ± 2.58	0.42 ± 0.09	7.87 ± 0.06	1.17 ± 0.91	20.06 ± 1.06	4.82 ± 0.96	36.89 ± 0.45	7.62 ± 0.61	56.11 ± 0.10	16.21 ± 2.50	59.60 ± 0.60	11.76 ± 1.36
∑T_∆_/°C·d		7.15		9.19		11.23		13.27		15.31		17.35	
		t_1_ = 11 h, t_2_ = 7 h; T_d_ = 24.29 °C		t_1_ = 9 h, t_2_ = 9 h; T_d_ = 23.88 °C		t_1_ = 7 h, t_2_ = 11 h; T_d_ = 23.46 °C		t_1_ = 5 h, t_2_ = 13 h; T_d_ = 23.04 °C		t_1_ = 3 h, t_2_ = 15 h; T_d_ = 22.63 °C		t_1_ = 1 h, t_2_ = 17 h T_d_ = 22.21 °C	
**H93S**	T_c_ = 23.0 °C T_Δ2=_2.0 °C	1.53 ± 0.45	1.01 ± 0.26	2.03 ± 0.27	0.85 ± 0.19	7.40 ± 0.88	1.91 ± 0.39	8.66 ± 1.71	2.55 ± 0.50	14.59 ± 0.13	5.68 ± 1.98	20.63 ± 0.54	5.03 ± 1.11
∑T_∆_/°C·d		4.08		5.25		6.42		7.58		8.75		9.92	
**PA64S**	T_c_ = 24.0 °C T_Δ2=_3.0 °C	5.86 ± 1.18	2.62 ± 0.64	10.12 ± 1.93	3.11 ± 0.84	16.25 ± 0.44	4.01 ± 0.26	18.38 ± 1.52	6.59 ± 1.41	32.75 ± 2.18	8.01 ± 0.45	42.33 ± 0.55	10.80 ± 0.45
∑T_∆_/°C·d		6.13		7.88		9.63		11.38		13.13		14.88	

T_Δ_ is the value of the ELT; T_c_ is the value of the CSIT; PFP is the pollen fertility percentage; SSP is the self-seed setting percentage; ΣT_Δ_ is the value of the cumulative ELT, calculated according to the formula ΣT_Δ_ = (T_Δ_*t_2_ *7 d)/24 h (for t_2_, see Table 2); and T_d_ is the DAT calculated according to Table 2 via the following formula T_d_ = {6 h*25 °C + t_1_*26 °C + t_2_*(T_c_-T_Δ_)}/24 h. All the data are expressed as the means ± the standard errors

At the same temperature, because the CSIT of PA64S was 24.0 °C, which was different from that of H93S (23.0 °C), the corresponding T_Δ_ was always 1.0 °C higher than that of H93S; i.e., T’_Δ1_ = 3.5 °C and T’_Δ2_ = 3.0 °C. As shown in Table 3, the DATs of the two treatments at t_1_ = 11 h and t_2_ = 7 h were higher than the CSIT of PA64S, leading to fertility restoration, with PFPs of 5.27% and 5.86% and SSPs of 0.42% and 2.62%, respectively. In the other treatments, all the DATs were lower than the CSIT of PA64S, with significantly increased PFP and SSP.

**Fig. 1:**
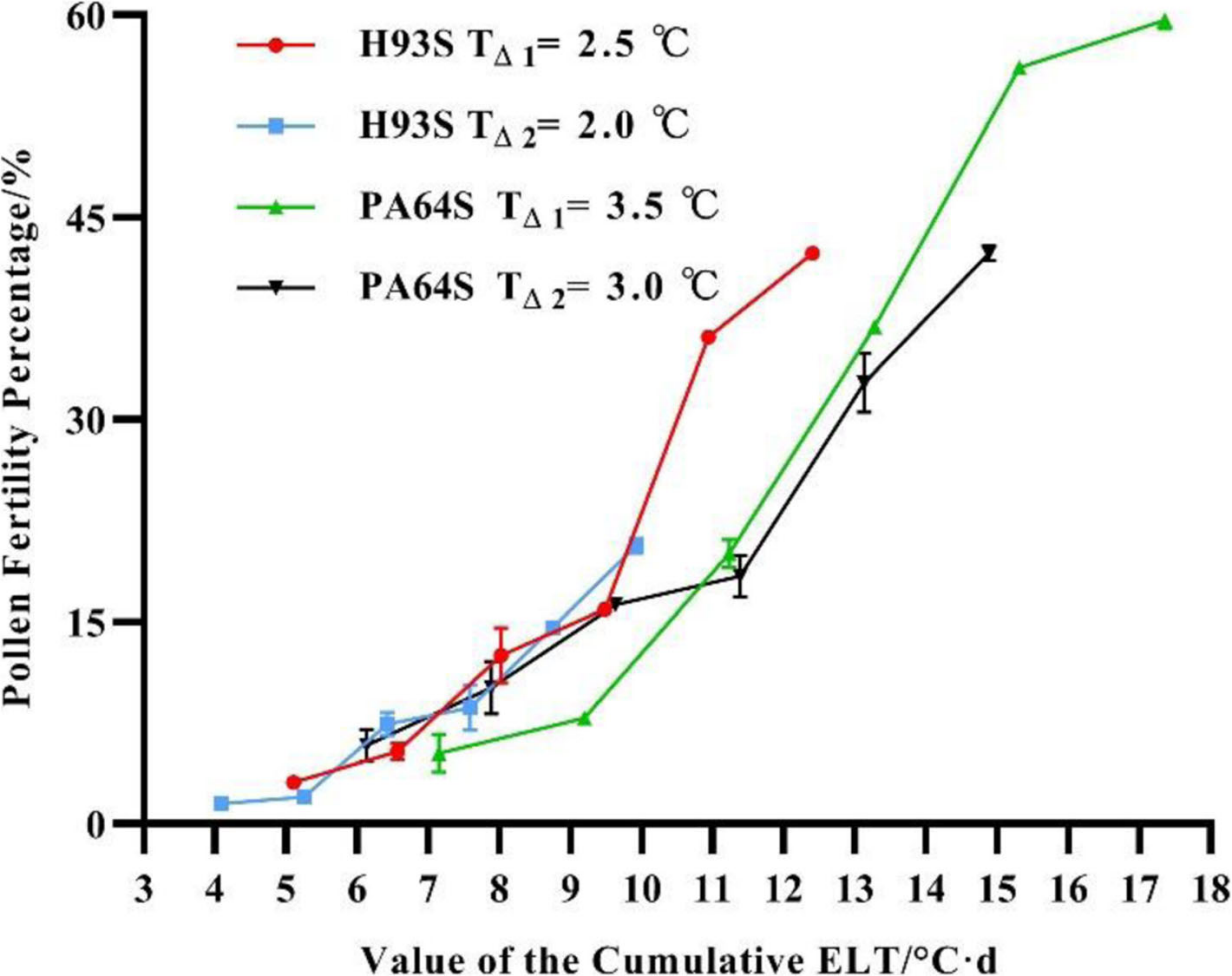
Pollen fertility percentage of H93S and PA64S after 7 d of treatment under different ΣT_Δ_ conditions. The actual data for all the ΣT_Δ_ values are shown in Table 3

On the basis of the results shown in Table 3, the PFP change trends for H93S and PA64S with the ΣT_Δ_ were plotted (Fig. 1). The graph indicates that as the ΣT_Δ_ increased, the PFPs gradually tended to increase, suggesting that the fertility transition of two-line male sterile rice is affected primarily by the cumulative ELT, which supports our hypothesis of the effect of cumulative ELT.

### Study of the Number of ELT-Response Days

**Fig. 2:**
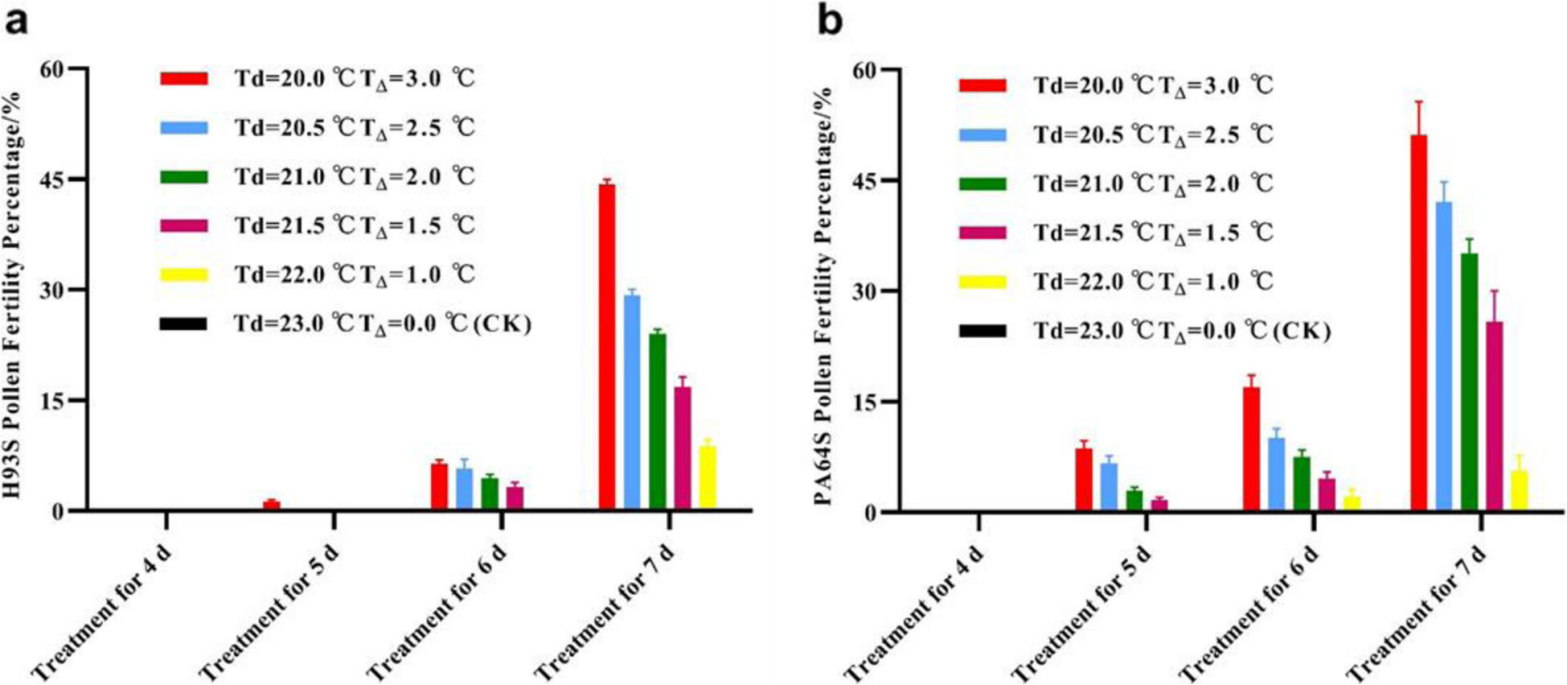
Pollen fertility of H93S and PA64S with different treatment days and different T_Δ_ values with a 13.5 h photoperiod. a Pollen fertility of H93S. b Pollen fertility of PA64S (Table 4 includes the actual data collected)

The results confirmed that there is an effect of cumulative ELT on the fertility transition of H93S as well as PA64S, which requires a certain number of ELT-response days or a certain ΣT_Δ_. Thus, we evaluated the number of ELT-response days (Table 4), and the results showed that at T_Δ_ = 0 °C (control), after 4–7 d of treatment, H93S and the control were sterile, with a PFP of 0%. At T_Δ_ = 1.0 °C, after 7 d of treatment, the PFPs of H93S and PA64S were 8.79% and 5.63%, respectively. At T_Δ_ = 1.5 °C, 2.0 °C, or 2.5 °C, the fertility transition initiated on day 6 after treatment for H93S and on day 5 for PA64S. Similarly, at T_Δ_ = 3.0 °C, for H93S, fertility transition occurred on day 5, with a PFP of 1.25%, which peaked on day 7 at 44.30%, while that of PA64S was 8.68% on day 5 and peaked at 51.22% on day 7. Figure 2 shows that as T_Δ_ and the number of treatment days increased, the pollen fertility of H93S and PA64S increased accordingly, even under the same number of treatment days, the fertility also increased along with the increasing T_Δ_.

**Table 4:** PFPs (%) of H93S and PA64S with different treatment days and different T_Δ_ values under a 13.5 h photoperiod

Treatment days (d)	T_Δ_ = 3.0 °C		T_Δ_ = 2.5 °C		T_Δ_ = 2.0 °C		T_Δ_ = 1.5 °C		T_Δ_ = 1.0 °C		T_Δ_ = 0 °C (CK)	
	H93S T_d_ = 20.0 °C	PA64S T_d_ = 21.0 °C	H93S T_d_ = 20.5 °C	PA64S T_d_ = 21.5 °C	H93S T_d_ = 21.0 °C	PA64S T_d_ = 22.0 °C	H93S T_d_ = 21.5 °C	PA64S T_d_ = 22.5 °C	H93S T_d_ = 22.0 °C	PA64S T_d_ = 23.0 °C	H93S T_d_ = 23.0 °C	PA64S T_d_ = 24.0 °C
4	0	0	0	0	0	0	0	0	0	0	0	0
5	1.25 ± 0.27	8.68 ± 1.05	0	6.61 ± 1.08	0	2.81 ± 0.43	0	1.62 ± 0.40	0	0	0	0
6	6.37 ± 0.54	17.03 ± 1.62	5.80 ± 1.22	10.08 ± 1.31	4.50 ± 0.46	7.54 ± 0.95	3.25 ± 0.65	4.60 ± 0.88	0	2.11 ± 0.95	0	0
7	44.30 ± 0.72	51.22 ± 4.46	29.24 ± 0.84	42.11 ± 2.73	24.03 ± 0.60	35.11 ± 1.99	16.86 ± 1.36	25.82 ± 4.20	8.79 ± 0.90	5.63 ± 2.10	0	0

T_Δ_ is the value of the ELT (see Table 2); T_d_ is the value of DAT, and T_d_ = T_c_-T_Δ_. All the data are expressed as the means ± the standard errors

## Discussion

### Importance of Determining the Precise CSIT

The CSIT of a PTGMS rice line must be determined before the line is released for actual application. In China, the CSIT is determined according to a specific protocol, i.e. NY/T1215–2006 (Sun et al. 2006) or local governmental regulations. The CSIT is a very important indicator for determining the location and period for hybrid seed production or self-propagation (Chen et al. 2011). However, to study the effect of cumulative ELT, this measurement of CSIT is not precise enough, because, according to NY/T1215–2006, the CSIT is determined by only ten treatments (24 °C and 28 °C under 11.5 h and 12.5 h photoperiods, and 23 °C, 24 °C, and 28 °C under 13.5 h and 14.5 h photoperiods), for which the intervals between the temperature settings are too large. For this is the reason, we added four treatments (Table 1) of which the temperatures are close to the original CSIT for H93S and PA64S. In addition, the CSIT of PA64S, which was identified as T_c_ = 23.3 °C when it was released (Luo et al. 1992), drifted upward and once reached to 24.2 °C (Lei et al. 2014) and was determined to be 24.0 °C under a 13.5 h photoperiod in this study.

To determine the effect of cumulative ELT, we estimated the value of ELT very precisely via T_Δ_ = T-T_c_. Indeed, if the CSIT is not very precise, the estimates of T_Δ_ deviate considerably.

### Photo-Thermo Response Characteristics of the Fertility Transition and its Practical Values

The results of the present study show that H93S is a PTGMS line with a CSIT of 23.0 °C under 13.5 h to 14.5 h photoperiods and a CSIT of 24.0 °C under a 12.5 h photoperiod, and its photo-thermo-response characteristics are consistent with the interaction model between day length and temperature (He et al. 2007). The CSIT not only changes with generation but also is associated with the photoperiod. As the light duration increases, the CSIT decreases, and vice versa (Tang et al. 2010). The photo-thermo-response characteristics of this type of sterile line are conducive to increased winter generation breeding in Hainan Province, China. During winter, the day length is short, resulting in concurrent increases of the CSIT, which is sufficient to expand the temporal and spatial ranges for self-propagation of two-line male sterile rice. As shown in Table 1, under an 11.5 h photoperiod and 23.0 °C, H93S showed a higher fertility transition with a PFP of 50.51%; however, very few pollen grains were fertile at 24 °C. This differed from the results of PA64S, where the fertility transition appeared higher at 24 °C under an 11.5 h photoperiod with a PFP of 78.31%. Empirically data have shown that, compared with the traditional procedure, the sowing date of PTGMS rice (e.g., H93S) can be postponed by approximately 10 d (in late November) in some parts of Hainan Province, such as Sanya (109.42°N, 18.39°E), Lingshui (109.95°N, 18.57°E), and Ledong (109.05°N, 18.65°E), such that a normal fertility transition can be achieved at a later time, resulting in a higher reproductive coefficient because the temperature during the sensitive period was slightly higher than the CSIT and the temperature during the subsequent heading and filling stages was more conducive to reproductive growth of rice.

### The Cumulative ELT and Response Days

In the present study, after exposure to a certain ΣT_Δ_ for a specific number of days, the fertility of H93S and PA64S could be restored. When the treatment lasted for 7 d, the fertility restoration of the male sterile line was the best with the greatest pollen fertility. The fertility transition of the male sterile line is positively correlated with ΣT_Δ_. The fertility of the male sterile line increased with the increasing ΣT_Δ_, which is consistent with the findings of previous studies. Wang et al. (2011b) reported that the fertility of P88S at 26.0 °C–28.0 °C is determined by the absolute value of the temperature and the effect of cumulative the temperature. Both Wang et al. (2011a) and Peng et al. (2016) demonstrated that the genetic background and CSIT of the fertility transition in a sterile line are correlated with its sensitivity to sterility at low temperatures. On the basis of our results (Table 4), we can infer that the minimal ΣT_Δ_ of H93S is 15.0 °C·d calculated as ΣT_Δ_ = T_Δ_ (3.0 °C) * 5 d or T_Δ_ (2.5 °C) * 6 d. However, the results of the 12 ELT treatment combinations (Table 3; Fig. 1) showed that the ΣT_Δ_ values were all lower than 15.0 °C·d ranging from 4.08 °C·d to 14.88 °C·d, thus enabling fertility transitions in both H93S and PA64S. As shown in Table 3, the number of treatment days was 7 d, and fertile pollen grains were observed for values as low as ΣT_Δ_ = 4.08 °C·d. Moreover, fertile pollen grains were observed on day 6 with a ΣT_Δ_ = 15.0 °C·d (Table 4) suggesting that the fertility transition of H93S requires a certain ΣT_Δ_, as well as a certain number of response days. A lower ΣT_Δ_ corresponds to a lower number of response days. Based on this study, the fertility transition of the sterile line requires only a certain period of low temperature exposure every day, and other periods can be higher than the CSIT. This also suggests that H93S has a longer duration of low temperature exposure and that if ALT lasts 4–5 d, no fertility transition occurs; thus it is safe and reliable for seed production.

### Proposal for the Hypothesis of the Effect of Cumulative ELT

**Fig. 3:**
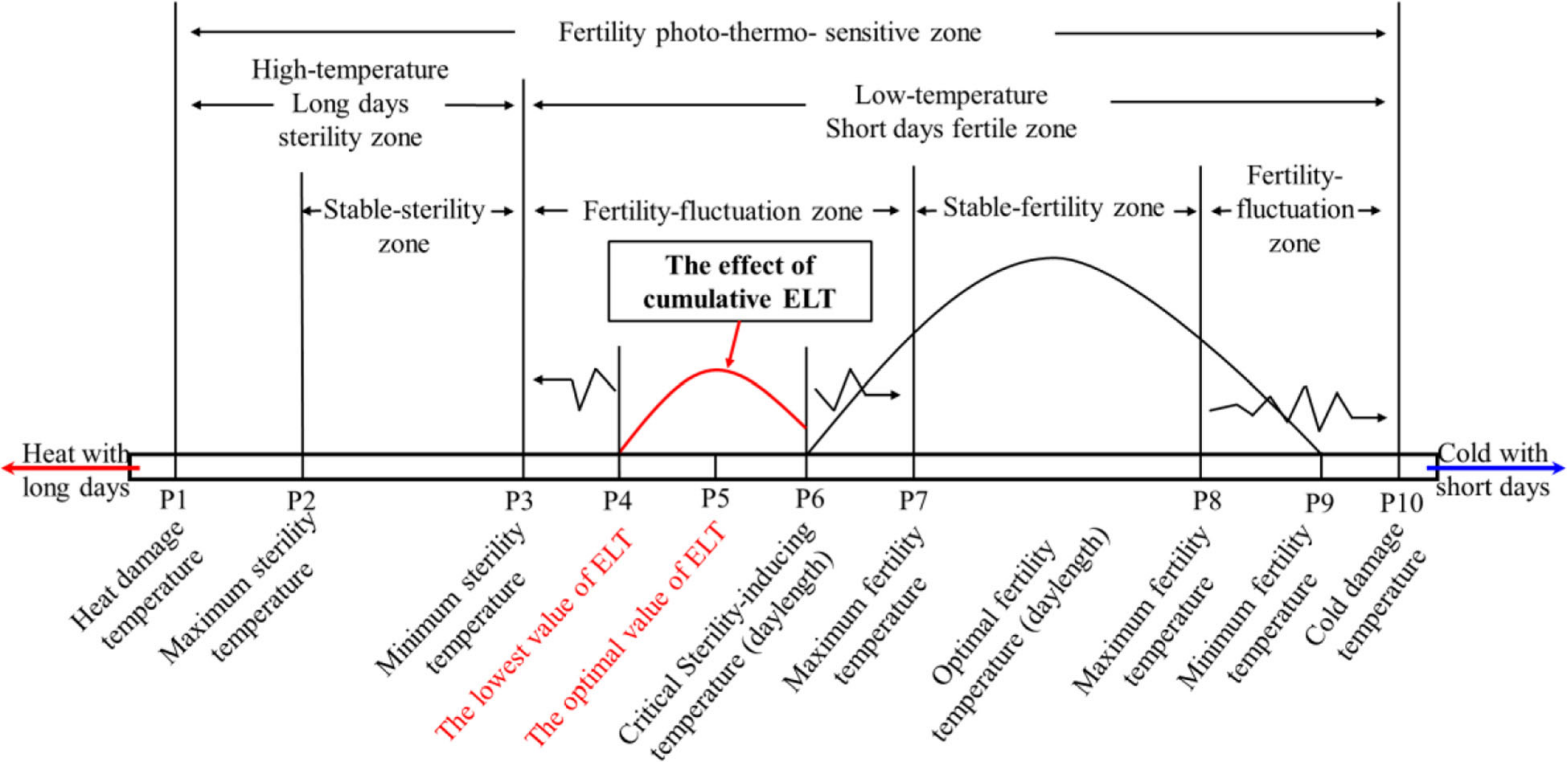
General scheme of the fertility transition for two-line male sterile rice. The position of red-colored section (from P4 to P6) was modified (added) according to Yao et al. (1995). The horizontal bars represent temperature and day length. The zig-zag lines and the red arrow indicate that the interval lengths between P4 and P3, P6 and P7, P8 and P9, and P4 and P6, respectively, fluctuate due to the changes of photo-thermo-conditions, which constitute the “sterility-fluctuation zone”. P6 indicates the CSIT (daylength). The transition from P6 to P9 corresponds the stable-fertility zone under optimal photo-thermo-conditions while the transition from P3 to P2 corresponds to the stable-sterility zone due to high temperatures and long days. The transition from P9 to P10, or from P2 to P1 is not recommended for self-pollination (propagation) or hybrid seed production because of the extremely low or high temperature, respectively. The transition from P4 to P6, which was not reported by Yao et al. (1995), is the zone of the effect of cumulative ELT which may explain the fertility restoration observed in two-line male sterile rice under ALT (The DAT of the ALT is higher than the CSIT of two-line male sterile rice, but the nighttime temperature is lower than the CSIT)

The main point of this study is that the fertility-sensitive period of PTGMS does not necessarily require the DAT to be lower than CSIT for 4–7 d to restore fertility. The fertility transition of the sterile line requires only a certain period of low temperature exposure every day, and the temperature of other periods can be higher than the CSIT. In the present study, after the treatment was applied for seven consecutive days for 7 h at temperatures that were 2.0 °C higher than the T_Δ_ each day (under a 13.5 h photoperiod, and during other periods, the temperature could be higher than the CSIT), the fertility of H93S was restored, which was also the case for PA64S. This phenomenon supports our hypothesis of the effect of cumulative ELT. Based on this hypothesis, we modified the general fertility transition scheme for the photo-thermo-sensitive male sterile line proposed by Yao et al. (1995), as shown in Fig. 3. Under this new scheme, when the actual (day length) temperature is higher than the fertility transition-critical (day length) temperature, the two-line male sterile rice is sterile. During the fertility-fluctuation period (the period encompassing the stable sterility temperature to the CSIT), the cumulative ELT affects the fertility status, and a certain ΣT_Δ_ can restore fertility of the two-line male sterile rice. We hypothesize that the effect of the cumulative ELT indicates that the value of the ELT is a key factor affecting the fertility transition within the fertility-fluctuation zone. When the DAT was higher than the CSIT but there was nighttime ALT that reached the lowest ELT, fertility was restored. These findings may explain why hybrid seed production of two-line male sterile rice failed when ALT occurred and supplement the knowledge concerning the fertility transition.

## Conclusions

The main cause of fertility transition is not the DAT being lower than the CSIT but instead is the effect of cumulative ELT. In two-line male sterile rice breeding, the relationship between the DAT of the fertility-sensitive period and the CSIT should not be considered alone. The effects of day length, ALT conditions, and continuous response days should also be considered. As the global climate warms, and the temperature difference between day and night is increasing. The DAT is becoming higher than the CSIT, and ALT together with a night temperature lower than the CSIT frequently occur, which introduces challenges to two-line hybrid seed production because two-line male sterile rice can transition to fertile during the fertility-sensitive period (which is usually from stage IV to stage VI of panicle differentiation), resulting self-pollination. The results showed that fertility transition is not only related to the relationship between the DAT and CSIT but also to the effect of the cumulative ELT. Additionally, a long duration of low-temperature exposure plays an important role in the fertility transition. The effect of cumulative ELT serves as another scientific and rigorous factor for breeding and hybrid seed production and effectively changes in response to fluctuating global climatic conditions, reducing the risk and expanding the purity and yield of rice. The present study provides new insight into the fertility transition and breeding of two-line male sterile rice.

## Supplementary Information

**Additional file 1 Fig. S1**. Images of the facility used to apply the treatments described in the text. **a** and **b** Plants growing in precise constant temperature cold water baths for the constant temperature treatments listed in Table 3 and Table 4 with a 13.5 h photoperiod; **c** At the end of the treatment, tillers whose distance between the ligule of the flag leaf and that of the next leaf was approximately 1.0 cm were marked, and the plants were moved back to the field. **d** and **e** Plants were treated in artificial climate chambers for determining the CSIT. Table 1, Table 2, Table S1 Table S2 and Table S3 include the treatment settings. **f** and **g** Plants were treated in a large scale smart greenhouse for the study the effect of cumulative ELT by simulating ALT conditions. **h** The plants were ready to be transplanted back to the field after the treatments were applied. All the plants under treatment were during their fertility-sensitive period (at the young panicle differentiation IV to VI stage). **Fig. S2**. Pollen and anther morphology of fertility restoration and abortion of H93S and the control PA64S. The images show the **a**) Normal pollen and **b**) anther morphology of the fertility restoration of H93S (12.5 h, 23.0 °C). **c**) Aborted pollen and **d**) anther morphology of sterile H93S (11.5 h, 24.0 °C). **e**) Normal pollen and **f**) anther morphology of fertile PA64S (11.5 h, 23.0 °C). **g**) Aborted pollen and **h**) anther morphology of sterile PA64S (14.5 h, 23.0 °C). Scale bars are 100 μm (**a**, **c**, **e**, **g**) and 1 mm (**b**, **d**, **f**, **h**)**Table S1**. Light-temperature conditions during the day at T_∆1_ = 2.5 °C**. Table S2**. Light-temperature conditions during the day at T_∆2_ = 2.0 °C. **Table S3**. Temperature settings for the study of effective treatment days (photoperiod of 13.5 h).

## Data Availability

Not applicable.

## Abbreviations

PTGMS: Photo-thermo-sensitive genic male sterile
ALT: Abnormally low-temperature
DAT: Daily average temperature
CSIT: Critical sterility-inducing temperature
H93S: Hang93S
ELT: Effective low temperature
PGMS: Photoperiod-sensitive genic male sterile
ΣT_Δ_: The cumulative ELT
T_Δ_: The value of the ELT
PA64S: PeiAi64S
T_c_: The value of CSIT
T_d_: The value of DAT
T: Actual treatment temperature
I_2_-KI: 1% Potassium iodide solution
PFP: Pollen fertility percentage
SSP: Self-seed setting percentage
t_1_: Treatment time at 26.0 °C
t_2_: Treatment time at the temperature of T_c_-T_Δ_
