# Supplementary material for: Characteristics of Fertility Transition Response to the Cumulative Effective Low Temperature in a Two-Line Male Sterile Rice Cultivar
Source: Rice (N Y). 2021 Aug 3;14:71. doi: 10.1186/s12284-021-00514-8 (PMC8333178; doi:10.1186/s12284-021-00514-8)
Supplement: Supplementary file 1 — Additional file 1 Fig. S1. Images of the facility used to apply the treatments described in the text. a and b Plants growing in precise constant temperature cold water baths for the constant temperature treatments listed in Table 3 and Table 4 with a 13.5 h photoperiod; c At the end of the treatment, tillers whose distance between the ligule of the flag leaf and that of the next leaf was approximately 1.0 cm were marked, and the plants were moved back to the field. d and e Plants were treated in artificial climate chambers for determining the CSIT. Table 1, Table 2, Table S1 Table S2 and Table S3 include the treatment settings. f and g Plants were treated in a large scale smart greenhouse for the study the effect of cumulative ELT by simulating ALT conditions. h The plants were ready to be transplanted back to the field after the treatments were applied. All the plants under treatment were during their fertility-sensitive period (at the young panicle differentiation IV to VI stage). Fig. S2. Pollen and anther morphology of fertility restoration and abortion of H93S and the control PA64S. The images show the a) Normal pollen and b) anther morphology of the fertility restoration of H93S (12.5 h, 23.0 °C). c) Aborted pollen and d) anther morphology of sterile H93S (11.5 h, 24.0 °C). e) Normal pollen and f) anther morphology of fertile PA64S (11.5 h, 23.0 °C). g) Aborted pollen and h) anther morphology of sterile PA64S (14.5 h, 23.0 °C). Scale bars are 100 μm (a, c, e, g) and 1 mm (b, d, f, h)Table S1. Light-temperature conditions during the day at T∆1 = 2.5 °C. Table S2. Light-temperature conditions during the day at T∆2 = 2.0 °C. Table S3. Temperature settings for the study of effective treatment days (photoperiod of 13.5 h). [file 12284_2021_514_MOESM1_ESM.docx]

***Supplementary Materials***


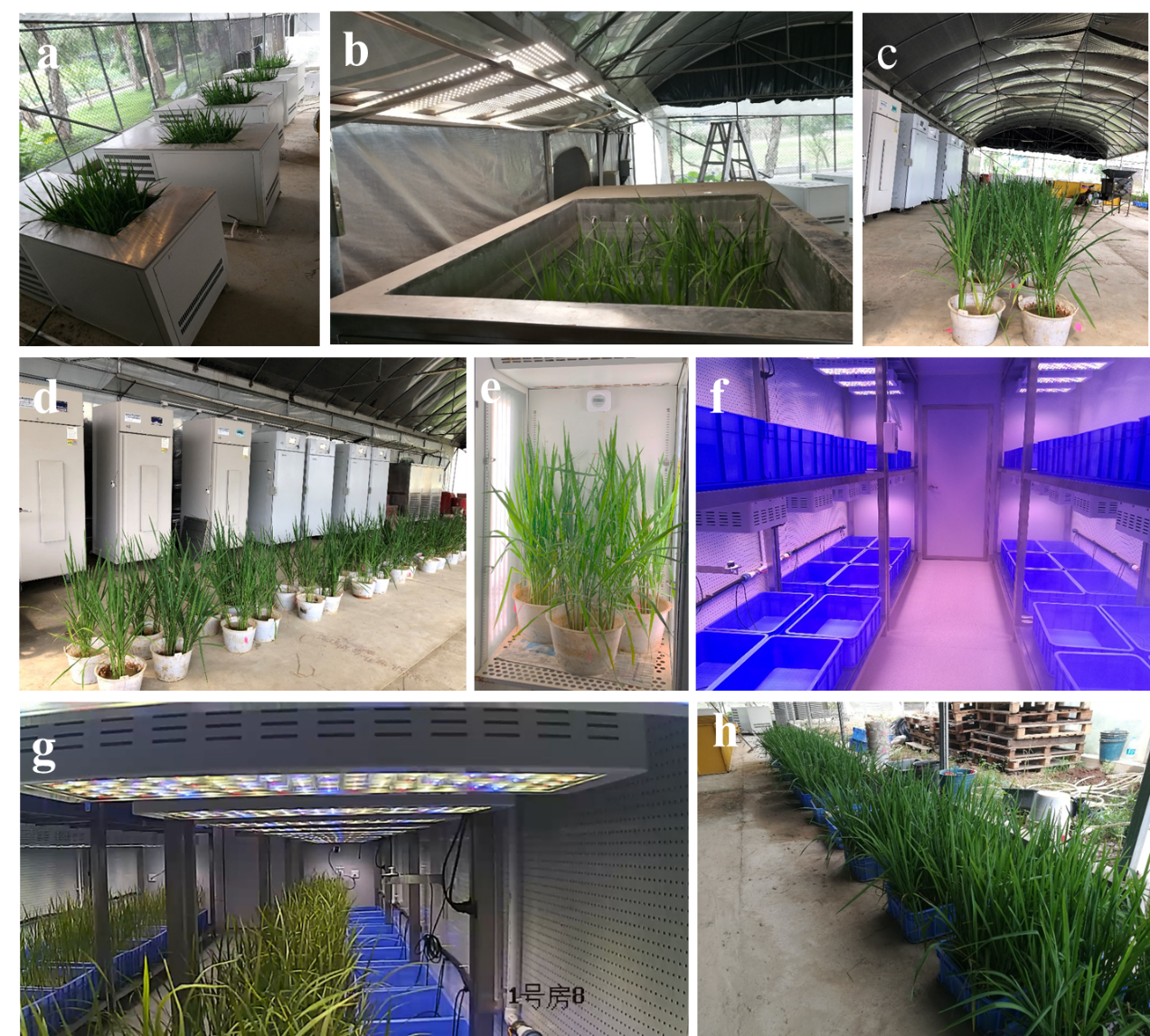


**Fig. S1** Images of the facility used to apply the treatments described in the text. **a** and **b** Plants growing in precise constant temperature cold water baths for the constant temperature treatments listed in Table 3 and Table 4 with a 13.5 h photoperiod; **c** At the end of the treatment, tillers whose distance between the ligule of the flag leaf and that of the next leaf was approximately 1.0 cm were marked, and the plants were moved back to the field. **d** and **e** Plants were treated in artificial climate chambers for determining the CSIT. Table 1, Table 2, Table S1 Table S2 and Table S3 include the treatment settings. **f** and **g** Plants were treated in a large scale smart greenhouse for the study the effect of cumulative ELT by simulating ALT conditions. **h** The plants were ready to be transplanted back to the field after the treatments were applied. All the plants under treatment were during their fertility-sensitive period (at the young panicle differentiation IV to VI stage)


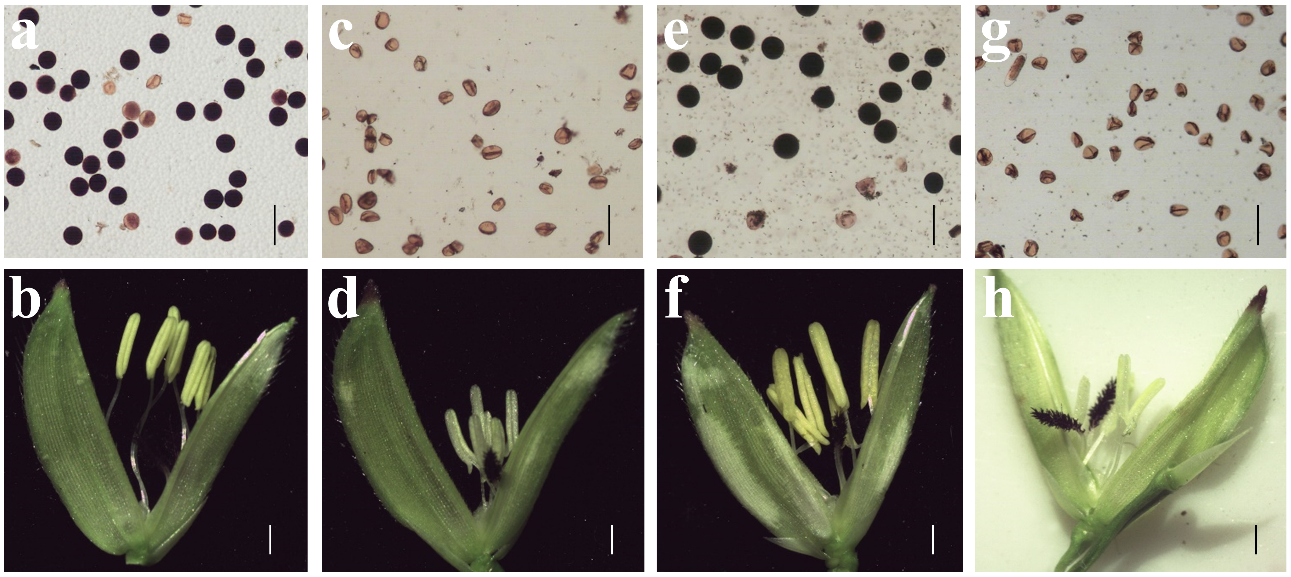


**Fig. S2** Pollen and anther morphology of fertility restoration and abortion of H93S and the control PA64S. The images show the **a**) Normal pollen and **b**) anther morphology of the fertility restoration of H93S (12.5 h, 23.0 °C). **c**) Aborted pollen and **d**) anther morphology of sterile H93S (11.5 h, 24.0 °C). **e**) Normal pollen and **f**) anther morphology of fertile PA64S (11.5 h, 23.0 °C). **g**) Aborted pollen and **h**) anther morphology of sterile PA64S (14.5 h, 23.0 °C). Scale bars are 100 μm (**a**, **c**, **e**, **g**) and 1 mm (**b**, **d**, **f**, **h**)

Table S1 Light-temperature conditions during the day at T_∆1_=2.5 °C

| DAT (°C) | Time period | Time (min) | Temperature (°C)  ) | Light (Lx) |
| --- | --- | --- | --- | --- |
| 24.15 | 06:00 - 08:00 | 120 | 25 | 5000 (06:30-08:00) |
|  | 08:00 - 09:00 | 60 | 25 | 20,000 |
|  | 09:00 - 12:00 | 180 | 26 | 20,000 |
|  | 12:00 - 18:00 | 360 | 26 | 20,000 |
|  | 18:00 - 20:00 | 120 | 26 | 5000 |
|  | 20:00 - 23:00 | 180 | 25 | 0 |
|  | 23:00 - 06:00 | 420 | 20.5 | 0 |
| 23.69 | 06:00 - 08:00 | 120 | 25 | 5000 (06:30-08:00) |
|  | 08:00 - 09:00 | 60 | 25 | 20,000 |
|  | 09:00 - 12:00 | 180 | 26 | 20,000 |
|  | 12:00 - 18:00 | 360 | 26 | 20,000 |
|  | 18:00 - 20:00 | 120 | 25 | 5000 |
|  | 20:00 - 21:00 | 60 | 25 | 0 |
|  | 21:00 - 06:00 | 540 | 20.5 | 0 |
| 23.23 | 06:00 - 08:00 | 120 | 25 | 5000 (06:30-08:00) |
|  | 08:00 - 11:00 | 180 | 25 | 20,000 |
|  | 11:00 - 12:00 | 60 | 26 | 20,000 |
|  | 12:00 - 18:00 | 360 | 26 | 20,000 |
|  | 18:00 - 19:00 | 60 | 25 | 5000 |
|  | 19:00 - 20:00 | 60 | 20.5 | 5000 |
|  | 20:00 - 06:00 | 600 | 20.5 | 0 |
| 22.77 | 06:00 - 08:00 | 120 | 20.5 | 5000 (06:30-08:00) |
|  | 08:00 - 12:00 | 240 | 25 | 20,000 |
|  | 12:00 - 17:00 | 300 | 26 | 20,000 |
|  | 17:00 - 18:00 | 60 | 25 | 20,000 |
|  | 18:00 - 19:00 | 60 | 25 | 5000 |
|  | 19:00 - 20:00 | 60 | 20.5 | 5000 |
|  | 20:00 - 06:00 | 600 | 20.5 | 0 |
| 22.31 | 06:00 - 08:00 | 120 | 20.5 | 5000 (06:30-08:00) |
|  | 08:00 - 12:00 | 240 | 25 | 20,000 |
|  | 12:00 - 15:00 | 180 | 26 | 20,000 |
|  | 15:00 - 17:00 | 120 | 25 | 20,000 |
|  | 17:00 - 18:00 | 60 | 20.5 | 20,000 |
|  | 18:00 - 20:00 | 120 | 20.5 | 5000 |
|  | 20:00 - 06:00 | 600 | 20.5 | 0 |
| 21.85 | 06:00 - 08:00 | 120 | 20.5 | 5000 (06:30-08:00) |
|  | 08:00 - 11:00 | 180 | 25 | 20,000 |
|  | 11:00 - 12:00 | 60 | 26 | 20,000 |
|  | 12:00 - 15:00 | 180 | 25 | 20,000 |
|  | 15:00 - 18:00 | 180 | 20.5 | 20,000 |
|  | 18:00 - 20:00 | 120 | 20.5 | 5000 |
|  | 20:00 - 06:00 | 600 | 20.5 | 0 |

DAT, daily mean temperature=(temperature×time)/(60 min×24 h).

Table S2 Light-temperature conditions during the day at T_∆2_=2.0 °C

| DAT (°C) | Time period | Time (min) | Temperature (°C)  ) | Light (Lx) |
| --- | --- | --- | --- | --- |
| 24.29 | 06:00 - 08:00 | 120 | 25 | 5000 (06:30-08:00) |
|  | 08:00 - 09:00 | 60 | 25 | 20,000 |
|  | 09:00 - 12:00 | 180 | 26 | 20,000 |
|  | 12:00 - 18:00 | 360 | 26 | 20,000 |
|  | 18:00 - 20:00 | 120 | 26 | 5000 |
|  | 20:00 - 23:00 | 180 | 25 | 0 |
|  | 23:00 - 06:00 | 420 | 21 | 0 |
| 23.88 | 06:00 - 08:00 | 120 | 25 | 5000 (06:30-08:00) |
|  | 08:00 - 09:00 | 60 | 25 | 20,000 |
|  | 09:00 - 12:00 | 180 | 26 | 20,000 |
|  | 12:00 - 18:00 | 360 | 26 | 20,000 |
|  | 18:00 - 20:00 | 120 | 25 | 5000 |
|  | 20:00 - 21:00 | 60 | 25 | 0 |
|  | 21:00 - 06:00 | 540 | 21 | 0 |
| 23.46 | 06:00 - 08:00 | 120 | 25 | 5000 (06:30-08:00) |
|  | 08:00 - 11:00 | 180 | 25 | 20,000 |
|  | 11:00 - 12:00 | 60 | 26 | 20,000 |
|  | 12:00 - 18:00 | 360 | 26 | 20,000 |
|  | 18:00 - 19:00 | 60 | 25 | 5000 |
|  | 19:00 - 20:00 | 60 | 21 | 5000 |
|  | 20:00 - 06:00 | 600 | 21 | 0 |
| 23.04 | 06:00 - 08:00 | 120 | 21 | 5000 (06:30-08:00) |
|  | 08:00 - 12:00 | 240 | 25 | 20,000 |
|  | 12:00 - 17:00 | 300 | 26 | 20,000 |
|  | 17:00 - 18:00 | 60 | 25 | 20,000 |
|  | 18:00 - 19:00 | 60 | 25 | 5000 |
|  | 19:00 - 20:00 | 60 | 21 | 5000 |
|  | 20:00 - 06:00 | 600 | 21 | 0 |
| 22.63 | 06:00 - 08:00 | 120 | 21 | 5000 (06:30-08:00) |
|  | 08:00 - 12:00 | 240 | 25 | 20,000 |
|  | 12:00 - 15:00 | 180 | 26 | 20,000 |
|  | 15:00 - 17:00 | 120 | 25 | 20,000 |
|  | 17:00 - 18:00 | 60 | 21 | 20,000 |
|  | 18:00 - 20:00 | 120 | 21 | 5000 |
|  | 20:00 - 06:00 | 600 | 21 | 0 |
| 22.21 | 06:00 - 08:00 | 120 | 21 | 5000 (06:30-08:00) |
|  | 08:00 - 11:00 | 180 | 25 | 20,000 |
|  | 11:00 - 12:00 | 60 | 26 | 20,000 |
|  | 12:00 - 15:00 | 180 | 25 | 20,000 |
|  | 15:00 - 18:00 | 180 | 21 | 20,000 |
|  | 18:00 - 20:00 | 120 | 21 | 5000 |
|  | 20:00 - 06:00 | 600 | 21 | 0 |

DAT, daily mean temperature=(temperature×time)/(60 min×24 h).

Table S3 Temperature settings for the study of effective treatment days (photoperiod of 13.5 h)

| T_Δ_ (°C) | Treatment temperature for H93S (Tc=23.0 °C) | Treatment temperature for PA64S (Tc=24.0 °C) | Treatment days (d) |
| --- | --- | --- | --- |
| T_Δ_=0 (CK) | Td=23.0 | Td=24.0 | 4 |
|  |  |  | 5 |
|  |  |  | 6 |
|  |  |  | 7 |
| T_Δ_=1.0 | Td=22.0 | Td=23.0 | 4 |
|  |  |  | 5 |
|  |  |  | 6 |
|  |  |  | 7 |
| T_Δ_=1.5 | Td=21.5 | Td=22.5 | 4 |
|  |  |  | 5 |
|  |  |  | 6 |
|  |  |  | 7 |
| T_Δ_=2.0 | Td=21.0 | Td=22.0 | 4 |
|  |  |  | 5 |
|  |  |  | 6 |
|  |  |  | 7 |
| T_Δ_=2.5 | Td=20.5 | Td=21.5 | 4 |
|  |  |  | 5 |
|  |  |  | 6 |
|  |  |  | 7 |
| T_Δ_=3.0 | Td=20.0 | Td=21.0 | 4 |
|  |  |  | 5 |
|  |  |  | 6 |
|  |  |  | 7 |

T_Δ_ is the value of the ELT; Tc is the critical sterility-inducing temperature; Td is the value of DAT, Td=Tc-T_Δ_; natural light, ≈13.5 h
